# Retraction: CuS nanocrystal@microgel nanocomposites for light-regulated release of dual-drugs and chemo-photothermal synergistic therapy *in vitro*

**DOI:** 10.1039/d0ra90106g

**Published:** 2020-10-19

**Authors:** Laura Fisher

**Affiliations:** Royal Society of Chemistry Thomas Graham House, Science Park, Milton Road Cambridge CB4 0WF UK advances-rsc@rsc.org

## Abstract

Retraction of ‘CuS nanocrystal@microgel nanocomposites for light-regulated release of dual-drugs and chemo-photothermal synergistic therapy *in vitro*’ by Jie Sun *et al.*, *RSC Adv.*, 2016, **6**, 8722–8728. DOI: 10.1039/C5RA25870G

The Royal Society of Chemistry hereby wholly retracts this RSC Advances article due to concerns with the reliability of the data in the published article.

The TEM image in Fig. 1c contains duplicating features and inconsistencies within the image, which indicates that it has been manipulated.

Given the significance of the concern about the validity of the data, the findings presented in this paper are no longer reliable.

Jie Sun, Rijun Gui, Hui Jin, Na Li and Xiaojing Wang state that Fig. 1c has not been manipulated but have not been able to provide any satisfactory raw data to confirm its authenticity. The authors oppose the decision to retract.

Signed: Laura Fisher, Executive Editor, RSC Advances

Date: 21^st^ September 2020

## Supplementary Material

